# Techniques to study chimerism at the tissue level in humanized mice

**DOI:** 10.1177/03009858251386916

**Published:** 2025-11-21

**Authors:** Arin Cox, Esha Banerjee, Jillian Verrelle, Elinor Willis, Charles-Antoine Assenmacher, Giovanni Finesso, James C. Tarrant, Enrico Radaelli

**Affiliations:** 1 Penn Vet Comparative Pathology Core, University of Pennsylvania, Philadelphia, PA; 2 Altasciences Preclinical, Seattle, WA; 3 GSK, Collegeville, PA

**Keywords:** chimeric mice, humanized mice, immunohistochemistry, mouse models, xenografts

## Abstract

Understanding the origin, distribution, and biology of different cell populations in chimeric mice is critical for interpreting the pathological changes developed in these models. To this aim, the methodological work presented here illustrates the validation and application of a collection of labeling techniques to differentiate between specific mouse and human tissue/cell components in formalin-fixed paraffin-embedded samples from chimeric mice, especially those bearing human tumor and immune cells. First, broad approaches to identify cells of human origin using ubiquitous immunohistochemical targets such as HLA-A, Ku80, and human mitochondrial 60 kDa protein (hMito) were established using specimens from humanized mice and a human tissue microarray including both normal and neoplastic samples. Due to its crisp membranous immunoreactivity, HLA-A was the most useful marker for visual human cell identification; however, Ku80 and hMito may be suitable options when HLA-A is not expressed in the cells of interest. Importantly, using one or more of these markers provides a broad range of coverage for the vast majority of human-derived cells in chimeric mice. Second, tailored immunohistochemical or *in situ* hybridization methodologies to distinguish specific human or mouse cell subsets are presented, focusing on immune/inflammatory cells and human chimeric antigen receptor (CAR) T-cells. These diverse approaches are accompanied by descriptions of case examples highlighting practical diagnostic and experimental applications in the context of various humanized mouse models. While not comprehensive, this work represents a valuable starting reference for pathologists and investigators working with humanized mouse models and seeking to add spatial resolution to the complex landscape of chimeric tissues.

Chimeric mice bearing human-derived tissues have become an essential tool of translational and pre-clinical research, allowing for the examination of human systems in murine models. These chimeric models are made possible through the use of genetically engineered mice with severely compromised immune systems, such as NSG (NOD.Cg-*Prkdc*^scid^ *Il2rg*^tm1Wjl^/SzJ) or NOG (NOD.Cg-*Prkdc*^scid^ *Il2rg*^tm1Sug^/JicTac) mice and other comparable strains, which offer a permissive recipient environment for the incorporation of human cells.^1–6^ The generation of chimeric mice requires the presence of a cellular niche that can be filled by engrafted human cells. This can be accomplished by engrafting normal human tissues, such as liver, and also through the use of patient-derived tumor xenografts that take advantage of the immunocompromised environment to permit the growth of cancers collected from clinical samples.^13,40,46^ Chimeric mice engrafted with human immune systems are often referred to as “humanized” mice.^18^ The use of humanized mice allows for the examination of immune responses and testing of drug safety and efficacy, providing insights into in vivo responses.^10,15^ However, the immunocompromised mouse strains used for humanization may also provide a permissive environment for unregulated and unpredictable cellular interactions and behaviors.^1,28,30^ Most often, these disorders arise from the uncontrolled activation and proliferation of transplanted human immune cells, producing clinically significant disease in the mouse recipient that can result in the loss of research animals and the introduction of confounding factors in preclinical data interpretation.^1,28,30^ Identification of specific human or murine cell types becomes especially important in the context of these unpredictable cellular interactions when comparative pathologists are asked to assess the fate of the xenotransplanted human tissues/cells and differentiate between experimentally induced changes, treatment-related effects, and spontaneous lesions in chimeric mice.

Independent efforts from multiple comparative pathology groups have been instrumental in characterizing the effects of humanization in mice.^1,28,43,57,60^ However, previous research involving chimeric mice relied heavily on flow cytometry to prove the presence and composition of human cells in the mouse host. While convenient, this approach is devoid of any morphological context, preventing the mapping of spatial relationships of the human/mouse cellular interface.^6,39^ Other applications like mRNA reporters, galactosidase repeats, and Alu repeats are effective at identifying human cells using their innate expression patterns. Still, these methods, like flow cytometry, require dissociation of tissue samples and prohibit the use of those samples for morphology-based evaluations.^11,19,36,55,66^ And while nondestructive cell-tracing techniques exist, they require advanced planning and treatment of cells prior to engraftment to add markers such as fluorescent nanoparticles, lipid dyes, and fluorescent tags.^33,53,54^ Despite offering a nondestructive mechanism, not all of these tracing methods are resistant to tissue processing, which reduces their usefulness in tissues destined for pathological examination.^53^ In addition, although these techniques may be useful for studying cell localization, the preclinical testing of human cell therapies in mouse models requires the use of unmodified products for subsequent clinical translation.

Techniques that prioritize the preservation of tissue morphology and spatial arrangements of cells allow for the visual assessment of cellular interactions in intact tissues and the precise evaluation of tissue composition, cell distribution, and cell phenotype, which are vital for understanding the chimeric tissue microenvironment. To this aim, the methodological work presented here describes several labeling strategies to differentiate between specific mouse and human cellular components in formalin-fixed paraffin-embedded tissues from humanized mice. These validated techniques include broad approaches to distinguish virtually all cells of human origin using ubiquitous markers and more targeted methodologies to study specific subsets of interest in mouse or human cell populations, such as myeloid cells and human chimeric antigen receptor (CAR) T-cells. These strategies are accompanied by descriptions of case examples highlighting practical experimental and diagnostic applications.

## Materials and Methods

### Immunohistochemistry, Immunofluorescence, In Situ Hybridization, and Imaging

For immunohistochemistry (IHC), immunofluorescence (IF), and *in situ* hybridization (ISH), 5 µm thick paraffin sections were mounted on ProbeOn slides (15-188-51; Thermo Fisher Scientific, Waltham, Massachusetts). Chromogenic IHC and multiplex IF were performed as described elsewhere^42^ using a Leica BOND RXm automated platform combined with the Bond Polymer Refine Detection kit (DS9800; Leica, Wetzlar, Germany) for IHC or the OPAL Multiplex Detection Kit (NEL830001KT; Akoya Biosciences, Marlborough, Massachusetts) implemented onto a Leica BOND Research Detection System (DS9455) for IF. Briefly, after dewaxing and rehydration, sections were pretreated with the epitope retrieval BOND ER2 high pH buffer (AR9640; Leica) for 20 minutes at 98°C. Endogenous peroxidase was inactivated with 3% H_2_O_2_ for 10 minutes at room temperature. Nonspecific tissue–antibody interactions were blocked by incubating the sections for 30 minutes at room temperature with Leica PowerVision IHC/ISH Super Blocking solution (PV6122) for IHC or with the Akoya Biosciences Opal Antibody Diluent/Block solution (ARD1001EA) for IF. The same blocking solution also served as a diluent for the primary antibodies. Primary antibodies were incubated on the sections for 45 minutes at room temperature. A biotin-free, polymeric detection system consisting of horseradish peroxidase conjugated anti-rabbit (DS9800; Leica), anti-rat (MP7444; Vector Laboratories, Newark, California), or anti-mouse (PV6114; Leica) IgG was then applied for 25 minutes at room temperature. For IHC, immunoreactivity was then revealed with the diaminobenzidine chromogen reaction. Tissue sections were finally counterstained in hematoxylin, dehydrated in an ethanol series, cleared in xylene, and permanently mounted with a resinous mounting medium (Thermo Scientific ClearVue coverslipper). For IF, the sections were incubated with the Akoya Biosciences TSA reagents Opal 520 (FP1487001KT), 570 (FP1488001KT), and 690 (FP1497001KT) at a working concentration of 1/150 for 10 minutes at room temperature followed by Spectral DAPI nuclear counterstaining (FP1490) and mounting with Fluoromount-G (100-01; SouthernBiotech, Birmingham, Alabama). Negative controls were obtained by the replacement of the primary antibodies with irrelevant isotype-matched rabbit, rat, or mouse antibodies.

ISH was performed using a Leica BOND RXm automated platform using RNAscope technology. The RNAscope probes WPRE-O1 (450268) and WPRE-04 (540348) were used for the identification of the untranslated regions (UTRs) of the lentiviral vector used for human CAR T-cell transduction. For the chromogenic RNAscope assay, the specific detection kit (322150; ACD, Newark, California) was implemented with a Leica BOND RED Detection System (DS9390). For the multiplex fluorescent RNAscope assay, the specific ISH detection kit (322800; ACD) and the Automation Multiplex Detection Kit (NEL830001KT; Akoya Biosciences) were implemented with a Leica BOND Research Detection System (DS9455). For the chromogenic RNAscope assay, slides were counterstained in hematoxylin, air-dried, and permanently mounted with EcoMount (EM897L; Biocare, Pacheco, California). For the multiplex fluorescent RNAscope assay, slides were counterstained with DAPI (FP1490; Akoya Biosciences) and coverslipped with Fluoromount-G (100-01; SouthernBiotech). Technical positive and negative probes included murine *Ppib* (313918; ACD) and *dapB* (312038-C5; ACD), respectively. The Aperio Versa 200 instrument was used for image acquisition.

### Markers and Tissue Samples Selection for IHC, IF, and ISH Labeling

As described in previous works,^6,43,57^ rabbit antibodies targeting HLA-A (ab52922, Abcam, Cambridge, Massachusetts; IHC working dilution: 1/600) and human Ku80 (hKu80; 2180; Cell Signaling Technology, Danvers, Massachusetts; IHC working dilution: 1/100), as well as a mouse antibody against a 60 kDa protein on human mitochondria (hMito; MA5-12017; Invitrogen, Waltham, Massachusetts; IHC working dilution: 1/500), were employed as broad-spectrum immunohistochemical markers for identifying virtually all cells of human origin in chimeric mice.^9,10,13^ While other broad-spectrum antibodies have been used for this same purpose, including NUMA-1,^60^ a comprehensive testing of all the possible markers ubiquitously expressed in human tissues was beyond the scope of this work. In this context, the target’s cellular location and distribution were considered in the selection, with HLA-A as a membranous antigen, hKu80 as a nuclear antigen, and hMito as a cytoplasmic marker.^2,6,12,44^ The overall sensitivity of these 3 broad-spectrum markers was investigated using a 60-core tissue microarray (TMA) slide (NBP2-30233, Novus Biologicals, Centennial, Colorado) containing different types of normal (32 cores) and neoplastic (27 cores) human tissues with one carbon core. The human specificity of the selected broad-spectrum rabbit antibodies (ie, HLA-A and hKu80) was assessed on a standardized set of formalin-fixed, paraffin-embedded (FFPE) normal mouse tissues prepared from 3 experimentally naïve, adult male C57BL/6J mice. These normal mouse tissues were selected to match most of the normal human tissues in the TMA slide and included skin, esophagus, stomach, small intestine, large intestine, liver, kidney, urinary bladder, pancreas, salivary gland, lymph node, trachea, thyroid gland, lung, heart, skeletal muscle, brain, and testis. To avoid mouse-on-mouse labeling of endogenous immunoglobulins, the same standardized set of FFPE, normal mouse tissues from experimentally naïve, adult male NSG mice (*n* = 3) was used to test the mouse antibody targeting hMito. Spleens from NOD.Cg-*Prkdc*^scid^ *Il2rg*^tm1Wjl^ Tg (CMV-IL3, CSF2, KITLG)1Eav/MloySzJ (*n* = 3) or NOG-EXL (NOD.Cg-*Prkdc*^scid^ *Il2rg*^tm1Sug^ Tg (SV40/HTLV-IL3, CSF2)10-7Jic/JicTac) (*n* = 3) mice humanized with CD34+ hematopoietic stem cells from a previous study were also included to assess the ability of each marker to distinguish between neighboring human and mouse cells in chimeric samples.^65^

Narrow-spectrum markers were selected for specific cell subsets of hematopoietic origin (mainly lymphoid and myeloid populations) and human CAR T-cells (Supplemental Table S1). Their human and/or mouse specificity was validated on a standardized set of FFPE tissues, including pools of lymphoreticular tissues, and on occasion other organs, of human, immunocompetent mouse (ie, normal adult C57BL/6J or CD1 mice), and humanized mouse origin. Human lymphoid tissues included commercially sourced lymph nodes (NBP2-30183, NBP2-77810), tonsil (NBP2-30207), and bone marrow (NBP2-78017). The selected humanized mouse samples were characterized by the development of post-transplant conditions like chimeric myeloid cell hyperactivation syndrome or xenogeneic graft-versus-host disease (Supplemental Table S1) to emphasize the utility of these narrow-spectrum markers in diagnosing these conditions.

Probes and antibodies for detecting human CAR T-cells were tested in treated NSG mice. Two different ISH probes that target the UTRs of the lentiviral vector used for CAR transduction were tested (see the above description of the ISH materials and methods). For armored CAR T-cells expressing the human IL-18, IHC was employed to reveal IL-18-positive cells (ab243091; Abcam, IHC working dilution: 1/1000). In a few selected cases, the UTRs probe was combined with an undisclosed probe specific for the CAR binding target to demonstrate the spatial relationships of these 2 cell populations. Details concerning CAR engineering and targets remain confidential and cannot be disclosed at this time.

All mouse samples used in this work consisted of archival FFPE tissues retrieved from studies approved under the Institutional Animal Care and Use protocol #805789 of the University of Pennsylvania.

### TMA Analysis

A list of identifiable cell types was recorded for each tissue core in the TMA. Based on this list, a total of 31 cell populations were evaluated. If possible, normal cell types were paired with matching neoplastic cell types for comparison (eg, the sample of normal liver was compared to hepatocellular carcinoma), which was possible for 13 of 31 cell populations identified. Seventeen other cell types were only represented by normal samples. Osteosarcoma was available as a neoplastic sample and had no paired normal bone sample.

Human TMAs were immunolabeled with the 3 broad-spectrum antibodies HLA-A, Ku80, and hMito. IHC results were recorded in a semiquantitative manner, assigning each positive cell type within each tissue core an intensity grade 0-3 for each marker, encompassing negative (0), weak (1), moderate (2), and strong (3) signal intensity. Intensity was internally normalized between markers. When a cell type was represented in more than one tissue core and received different intensity scores, the modal score value was reported (Table 1). Cell populations within a core were considered positive when more than 60% of the cells belonging to that population were labeled with the antibody.

**Table 1.** Intensity scoring of HLA-A, Ku80, and hMito in cell populations represented in the human TMA core samples.

Cell Type	# Normal	# Neoplastic	HLA-A		Ku80		hMito	
			Normal	Neoplastic	Normal	Neoplastic	Normal	Neoplastic
Squamous epithelium	2^a^	4	3	3	3	3	3	3
Gastric mucosa	2	1	3	3	3	3	3	3
Small intestine mucosa	1	0	3	n/a	3	n/a	3	n/a
Large intestine mucosa	3	3	3	3	3	3	3	3
Salivary duct epithelium	1	0	2	n/a	3	n/a	3	n/a
Breast duct epithelium	1	1	0	0	3	3	3	3
Renal tubular epithelium	2	1	0	3	3	3	3	3
Urothelium	1	0	2	n/a	3	n/a	2	n/a
Thyroid epithelium	1	1	1	3	3	3	1	3
Pneumocytes	1	1	3	0	3	3	1	2
Gallbladder epithelium	1	1	3	3	3	3	3	3
Hepatocytes	1	1	3	2	2	3	3	3
Adrenal cortex	1	0	3	n/a	3	n/a	3	n/a
Lymphocytes	8	2	3	3	3	3	2	3
Vascular endothelium	24^b^	0	3	n/a	3	n/a	1	n/a
Vascular smooth muscle	5	0	0	n/a	3	n/a	1	n/a
Fibrovascular stroma	7	10	2	3	3	3	1	2
Bone (osteosarcoma)	0	1	n/a	3	n/a	3	n/a	3
Cardiac muscle	1	0	0	n/a	3	n/a	3	n/a
Skeletal muscle	1	0	0	n/a	2	n/a	2	n/a
GI smooth muscle	8	0	0	n/a	3	n/a	1	n/a
Endometrial glands	1	1	3	3	3	3	3	3
Myometrium	1	0	2	n/a	2	n/a	2	n/a
Germ cells	1	1	0	0	0	3	2	3
Interstitial cells	1	0	0	n/a	3	n/a	3	n/a
Sertoli cells	1	0	0	n/a	3	n/a	3	n/a
Endocrine pancreas	1	0	2	n/a	3	n/a	3	n/a
Peripheral ganglia	1	0	3	n/a	3	n/a	3	n/a
Cerebral neurons	1	0	0	n/a	2	n/a	2	n/a
Cerebellar granular layer	1	0	1	n/a	3	n/a	3	n/a
Cerebellar Purkinje cells	1	0	0	n/a	0	n/a	2	n/a

Abbreviations: TMA, tissue microarray; hHLA-A, human HLA-A; hKu-80, human Ku-80; hMito, human mitochondria; GI, gastrointestinal.

a hMito has only one normal squamous epithelium sample.

b hMito has only 15 endothelium samples; positivity was less than 60% in 9 samples.

The evaluation was conducted by 2 board-certified veterinary pathologists (A.C., E.R.). Pathologists resolved differing scores by consensus at the multiheaded microscope.

## Results

### Broad-Spectrum Markers for the Detection of Human Cells in Tissues From Chimeric Mice

The evaluation of the human specificity of the selected broad-spectrum markers confirmed diffuse IHC labeling in the human tissues in the TMA slides and engrafted human components in the humanized mouse samples. No immunoreactivity was detected in the normal mouse tissue or mouse components in the humanized mouse samples, with the exception of weak nuclear Ku80 immunolabeling in the adrenal cortex, renal tubules, and testes of C57BL/6 mice (Supplemental Figure S1a–c). Mast cells in the cervical lymph nodes of some C57BL/6 mice faintly labeled with HLA-A (Supplemental Figure S1d). No off-target or nonspecific labeling for hMito was noted in the NSG mouse tissues.

A summary of the results of broad-spectrum antibodies for human HLA-A, human Ku80, and hMito tested on the TMA of normal and neoplastic human tissues is presented in Table 1. Basic descriptive statistical parameters (median and interquartile range) are provided for those cell types represented by more than *n* = 3 tissue samples. This table is presented in the supplemental data (Supplemental Table S2).

**Figure 1.**
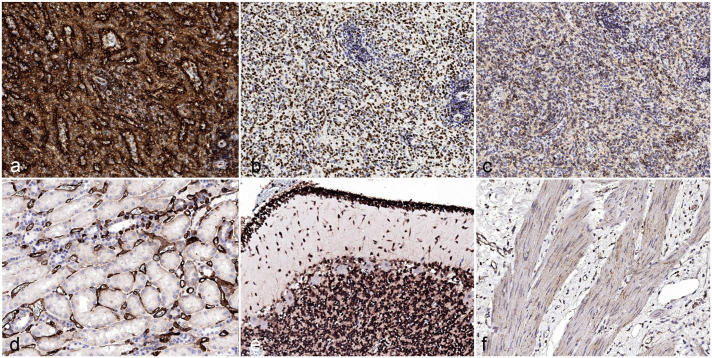
Comparison of positive (a, b, c) and negative (d, e, f) cell types for HLA-A (a, d), Ku80 (b, e), and hMito (c, f), human tissue microarray. (a) Lymph node. HLA-A expression is strong in all normal lymphocytes. (b) Lymph node. Ku80 labeling is variable in normal lymphocytes, but it strongly labels approximately 60% of cells. (c) Lymph node. hMito labeling is variable in normal lymphocytes, and the labeling intensity is weak to moderate. (d) Renal cortex. Tubular epithelium is negative for HLA-A, while the endothelium is strongly positive. (e) Cerebellum. The granular layers are strongly positive for Ku80; Purkinje cells are negative. (f) Gallbladder wall. Smooth muscle is weakly labeled for hMito, vascular endothelium is variably labeled, and fibrovascular stroma is not labeled.

HLA-A produced moderate to strong membranous labeling in most tissue samples (Fig. 1a, d). Out of 31 normal tissues evaluated in the TMA, HLA-A was positive in 19 tissues, and labeling intensity was graded as moderate or strong in 17 tissues. HLA-A expression was consistently positive in lymphocytes and most tissues of epithelial origin, with the exception of breast duct epithelium and renal tubular epithelium. HLA-A was negative in all types of muscle, testis (germinal epithelium, interstitial cells, and Sertoli cells), and central nervous system neurons. In neoplastic tissues, labeling for HLA-A was generally comparable with the paired normal tissue. Intriguingly, normal pneumocytes had strong labeling, while pulmonary adenocarcinoma was negative for HLA-A, and thyroid carcinoma had strong labeling for HLA-A compared with weak labeling in normal thyroid epithelium.

Human Ku80 produced moderate to strong nuclear labeling in virtually all samples, including all epithelial tissues, all types of muscle, all reproductive tissues except for developing sperm, and all nervous tissues except for Purkinje cells (Fig. 1b, e). Ku80 labeling in neoplastic tissue samples was similar or identical to normal tissues. Ku80 labeling was highly consistent between cores, with cells showing little to no variability in intensity (Supplemental Table S2).

Most tissues had variable labeling for hMito, and the label intensity and quality was variable between cores. Some cell types, such as vascular endothelium, did not reach the cut-off point for positivity (at least 60% labeling) in all tissue cores, which reduced the overall number of observations for hMito compared with HLA-A and Ku80. Labeling was weak (intensity score = 1) in many tissues, including thyroid and pulmonary epithelium, vascular endothelium, and smooth muscle. Some neoplastic tissue samples showed stronger labeling for hMito than normal tissues, including papillary thyroid carcinoma, pulmonary adenocarcinoma, and round cell neoplasms.

**Figure 2.**
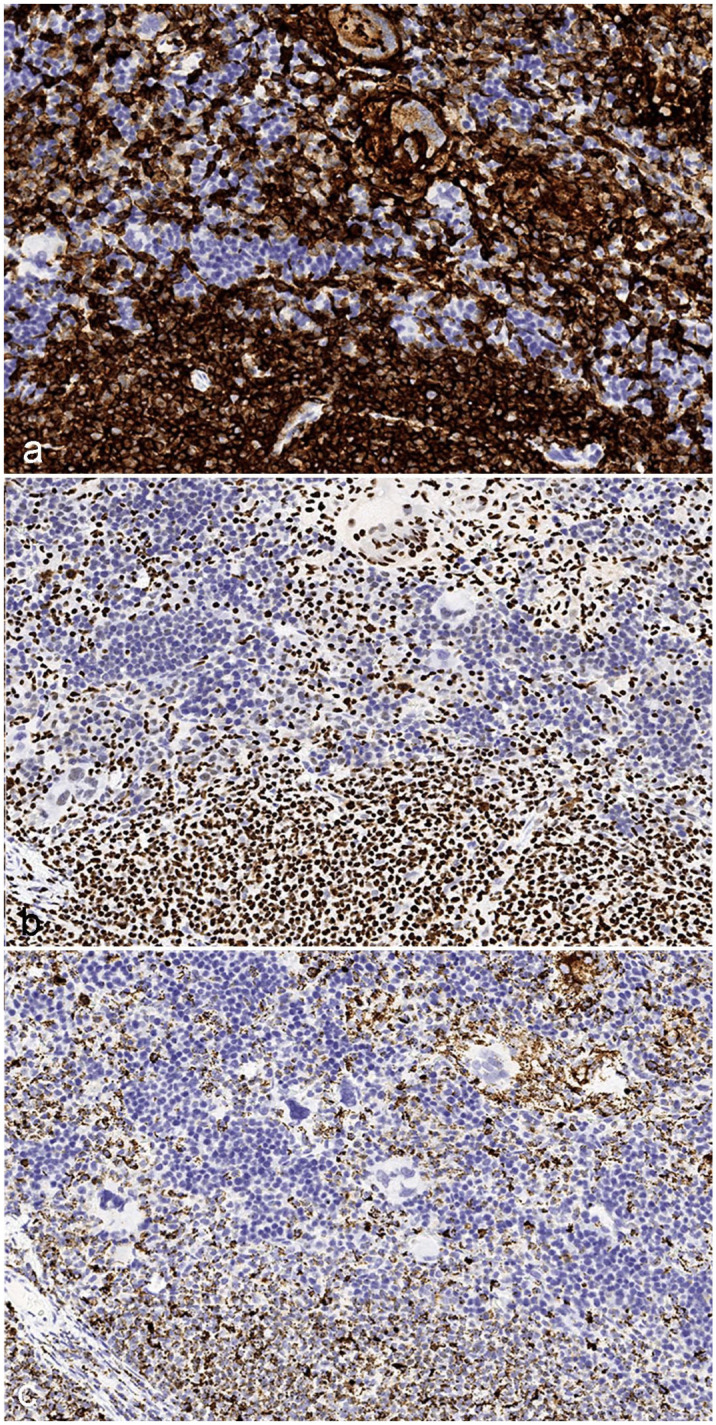
Comparison of marker pattern and quality for HLA-A, Ku80, and hMito, humanized NOG-EXL mouse, spleen. (a) HLA-A labeling is strong and membranous in human lymphocytes and myeloid cells. Cell borders are easily appreciated. (b) Ku80 labels only the nucleus. Individual nuclei are easily differentiated, but cell borders are unclear. (c) hMito labeling is granular to punctate, and it is difficult to delineate individual human cells and differentiate them from neighboring mouse cells.

Because of the diverse cell compartments recognized by each antibody, the IHC labeling pattern and quality diverged consistently across the different markers. Due to its crisp membranous immunoreactivity, human HLA-A allowed a clear distinction between neighboring human and mouse cells in humanized samples. However, HLA-A had a comparatively narrow spectrum of recognized tissues compared with Ku80 and hMito. Human Ku80 immunoreactivity was confined to the nuclear compartment, which created no distinct cellular margins but offered clear visualization of the nucleus. Due to its IHC pattern, mainly characterized by sparse cytoplasmic granules, hMito emerged as the least useful in delineating between adjacent human and mouse cells (Fig. 2a–c).

### Narrow-Spectrum Markers for the Distinction of Human and Mouse Immune/Inflammatory Cells in Tissues From Chimeric Mice

**Figure 3.**
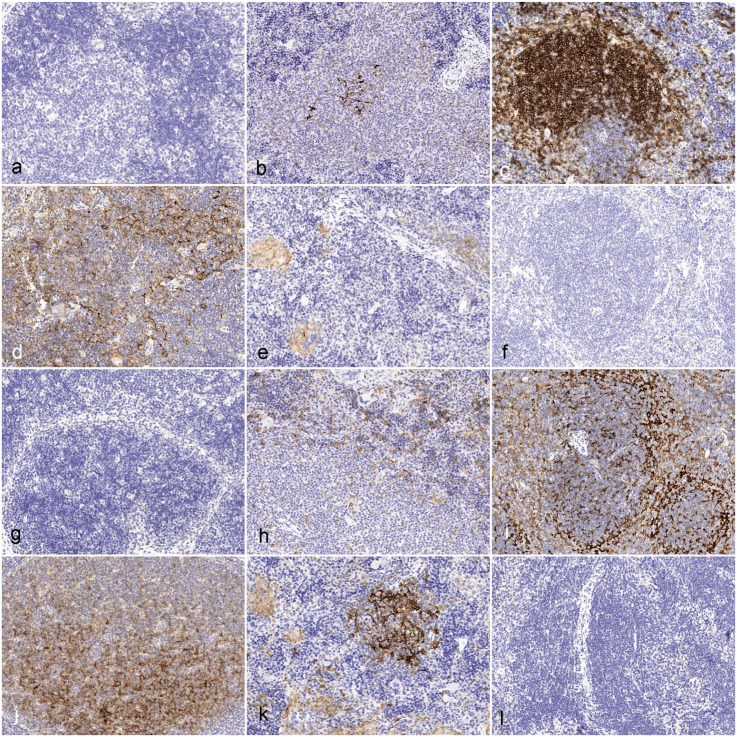
Comparison of murine CD40 (mCD40) (a, b, c), human CD40 (hCD40) (d, e, f), murine CD86 (mCD86) (g, h, i), and human CD86 (hCD86) (j, k, l) in human (a, d, g, j), humanized NOG-EXL mouse (b, e, h, k), and normal mouse (c, f, i, l) lymphoid tissues. (a) Human lymph node, negative for mCD40. (b) Humanized mouse, spleen. Dense cellular aggregate of negative mononuclear cells featuring a few central elements with strong mCD40 labeling. (c) Normal mouse, spleen. Cell aggregates label strongly with mCD40. (d) Human tonsil. Positive labeling for hCD40 in the follicle. (e) Humanized mouse, spleen. Small clusters of histiocytes, multinucleated giant cells, and a small aggregate of cells in the periarteriolar lymphoid sheath are positive for hCD40. (f) Normal mouse, spleen. No labeling for hCD40. (g) Human tonsil. There is no labeling with mCD86. (h) Humanized mouse, spleen. Small cell clusters in the red pulp are weakly to moderately positive for mCD86. (i) Normal mouse, spleen. Cells in the white pulp label strongly with mCD86. (j) Human lymph node. Cells in the white pulp are strongly positive for hCD86. (k) Humanized mouse, spleen. A cellular aggregate is strongly positive for hCD86, and giant cells are weakly positive. (l) Normal mouse, spleen. There is no labeling with hCD86.

For the narrow-spectrum antibodies, a comprehensive panel of human-specific or mouse-specific IHC markers was developed primarily to detect immune and inflammatory cell populations. This effort resulted in the identification of several matching antibodies where the complete lack of cross-reactivity between mouse and human tissues was validated using, in most cases, the combination of normal or reactive lymphoreticular tissues of human and mouse origin, including samples from immunocompetent mouse strains, and immunodeficient humanized mice as detailed in Supplemental Table S1. The panels enabled a granular investigation of different lymphoid and myeloid cell groups and lineages, including, for example, subpopulations of antigen-presenting cells expressing CD40 and CD86 (Fig. 3). The ability to distinguish between human and mouse myeloid markers was particularly informative in characterizing the immune response in humanized mice with conditions like xenogeneic graft-versus-host disease and chimeric myeloid cell hyperactivation syndrome (Supplemental Figures S2 and S3). A case example of an NSG mouse with human CAR T-cell-induced xenogeneic graft-versus-host disease is presented in Supplemental Figure S2. As confirmed via IHC, the affected mouse exhibited severe lymphohistiocytic salivary gland infiltrates comprised of human CAR T-cells expressing the human-specific CD45 LCA (leukocyte common antigen), murine macrophages positive for the mouse-specific macrophage marker F4/80, and murine antigen-presenting cells labeled with a mouse-specific CD86 antibody (Supplemental Table S1). A case example of chimeric myeloid cell hyperactivation syndrome in a NOG-EXL mouse humanized with human CD34+ hematopoietic stem cells is presented in Supplemental Figure S3. The mouse was clinically affected by paraparesis. While the mouse displayed the typical histopathological features of chimeric myeloid cell hyperactivation syndrome in the liver, spleen, and bone marrow, the microscopic changes affecting the lumbar spinal cord were nonspecific, being characterized by vague hypercellularity and axonal damage mainly involving the ventral funiculi. The subsequent application of IHC using a human-specific CD33 antibody (Supplemental Table S1) revealed a dense spinal cord infiltrate of CD33-positive human macrophages, confirming that this lesion has developed as part of the generalized unregulated activation and expansion of human myeloid cells. The use of human- and mouse-specific leukocyte markers was also instrumental for diagnosing neoplastic and non-neoplastic lymphoid proliferations in humanized mice, including Epstein-Barr virus-induced post-transplant lymphoproliferative disease, aberrant CAR T-cell proliferation, or spontaneous lymphoma/leukemia of mouse origin (Supplemental Figures S4–S6). An example of Epstein-Barr virus-induced post-transplant lymphoproliferative disease in a NOG mouse bearing a metastatic melanoma patient-derived xenograft is presented in Supplemental Figure S4. IHC using a human-specific CD138 antibody (Supplemental Table S1) confirmed the plasma cell differentiation of the atypical proliferation arising from Epstein-Barr virus-infected human B cells co-transplanted with the patient-derived tumor biopsy. An example of aberrant human CAR T-cell expansion in a treated NSG mouse is showcased in Supplemental Figure S5. The human CAR T-cell origin of this aberrant proliferation was confirmed by the diffuse expression of human-specific CD3 (Supplemental Table S1) by the atypical lymphoid cells as demonstrated via IHC. Finally, the diagnosis of a mouse mediastinal lymphoma arising spontaneously in an NSG mouse treated with CAR T-cells is presented in Supplemental Figure S6. In this case, the definitive diagnosis of a lymphoma of mouse origin was achieved by demonstrating diffuse expression of mouse-specific CD45 LCA in the atypical lymphoid infiltrate, while only scattered human CD45 LCA-positive lymphocytes, presumably circulating CAR T-cells, were represented within the tumor.

**Figure 4.**
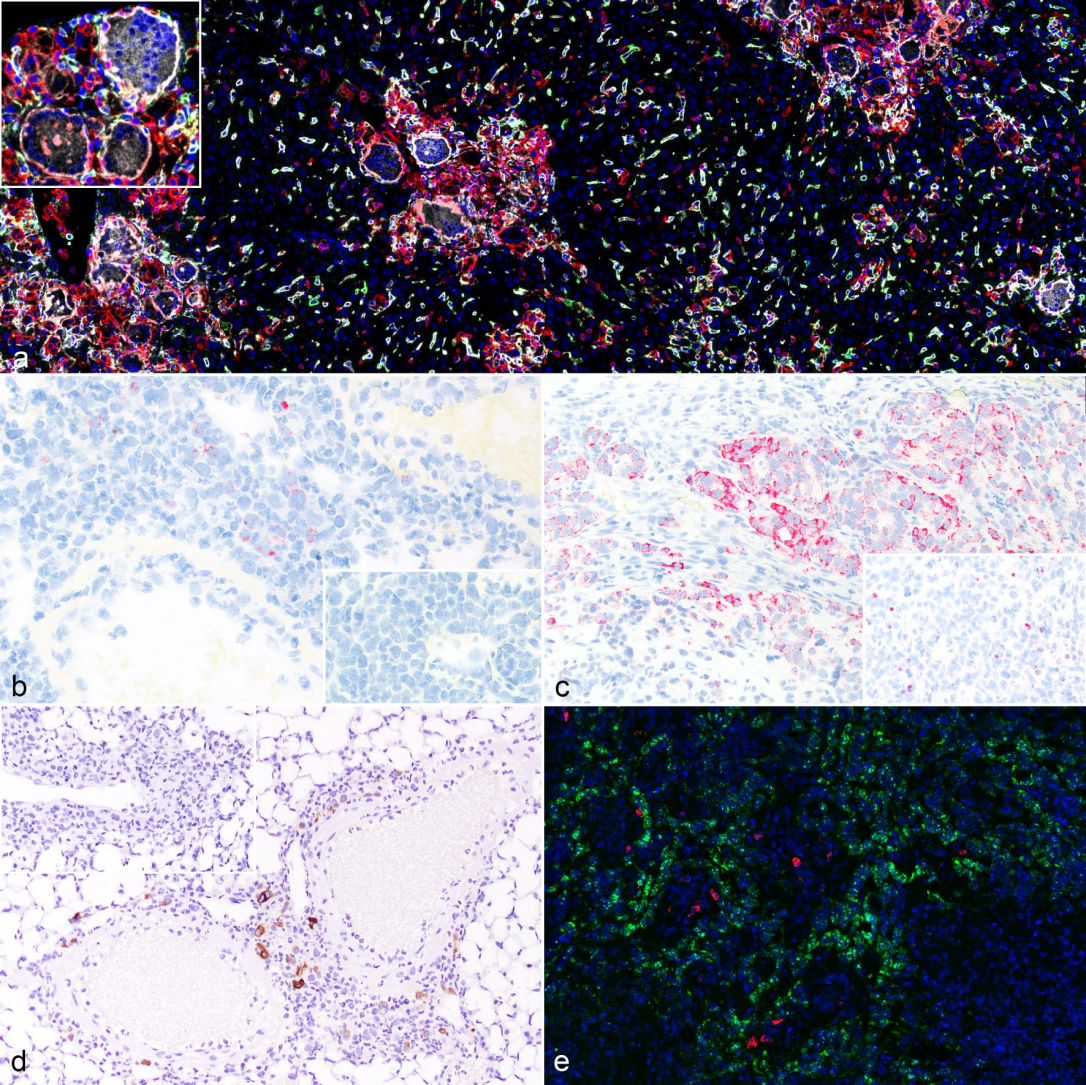
(a) Liver from a NOG-EXL mouse with chimeric myeloid cells hyperactivation syndrome. CD11b (white) is expressed in the infiltrating human multinucleated giant cells (inset) and reactive histiocytes that are identified by labeling with human CD45 LCA labeling (red). Similarly, CD11b (white) is expressed in the mouse Kupffer cells and macrophages that are identified by mouse CD45 LCA labeling (green). Multiplex immunofluorescence with mouse-specific CD45 LCA (green), human-specific CD45 LCA (red), human and mouse cross-binding CD11b (white), and DAPI counterstain (blue). (b) Lungs from an NSG mouse treated with human CAR T-cells. CAR expression in the perivascular pulmonary infiltrate of mononuclear cells is labeled with *in situ* hybridization (ISH) probes targeting the UTRs of the lentiviral vector used for transduction. Inset: Pulmonary infiltrate of untransduced human T-cells serve as a negative control. Chromogenic ISH for lentiviral UTRs. (c) Human tumor xenograft in an NSG mouse treated with human CAR T-cells. Both human T-cells for CAR T-cell manufacturing and the tumor cell line have been manipulated in vitro with lentiviral vectors. ISH for UTRs is positive in both tumor-infiltrating CAR T-cells and engrafted tumor cells, making it impossible to distinguish between the 2 populations. Inset: An example of UTRs expression in nontransduced CAR T-cells infiltrating a tumor xenograft. Chromogenic ISH for lentiviral UTRs. (d) Lungs from an NSG mouse treated with human armored CAR T-cells producing IL-18. Activated CAR T-cells in the perivascular pulmonary infiltrate of mononuclear cells are highlighted using immunohistochemistry for human IL-18. Inset: Perivascular infiltrate of nonarmored CAR T-cells that do not express IL-18. (e) Human tumor xenograft in an NSG mouse treated with human CAR T-cells. Multiplex fluorescent ISH assay allows the visualization of lentiviral UTRs in CAR T-cells (red) and the transcripts of the CAR-specific target in tumor cells (green). Multiplex fluorescent ISH with DAPI counterstain.

In addition, in the case of markers without species-specificity, the development and application of multiplex IF protocols strategically combining human-specific and mouse-specific broad-spectrum leukocyte markers, such as CD45 LCA, with the antibody of interest allowed for accurate determination of the composition of chimeric immune/inflammatory cell populations and infiltrates. For example, this approach was employed for the identification of CD11b expression in an NOG-EXL mouse suffering from chimeric myeloid cell hyperactivation syndrome. As the antibody used to detect CD11b recognized both the human and the mouse homolog targets (Supplemental Table S1), the distinction of CD11b expression in human and mouse macrophages required multiplex IF combining CD11b with both human- and mouse-specific CD45 LCA (Fig. 4a).

### Markers for the Identification of Human CAR T-Cells in Tissues From Treated Mice

During the preclinical testing of adoptive T-cell therapies, the fate of the delivered human T-cells within the immunodeficient mouse host can be easily traced using human-specific leukocyte markers like CD45 LCA or CD3, as shown in the above case examples (Supplemental Figures S5 and S6). However, in the case of human CAR T-cells, further distinction between T-cells with and without successful CAR transduction was evaluated using specific ISH probes recognizing the UTRs of the lentiviral vector used for CAR engineering in vitro. In the example shown in Fig. 4b, these probes were specifically employed to assess the actual CAR T-cell fraction contributing to infiltrates compatible with aberrant CAR T-cell proliferation in an NSG mouse. Both UTRs probes tested gave similar results, highlighting the coexistence of both CAR-transduced and nontransduced cells within these infiltrates.

CAR T cells are commonly administered in xenograft tumor models derived from cancer cell lines that have often been engineered in vitro with lentiviral vectors containing similar UTRs. As a result, UTRs do not constitute a reliable ISH target for distinguishing human CAR T cells infiltrating the tumor xenograft, since UTRs are nonspecific sequences present in all lentiviral constructs and thus are also expressed by the transduced cancer cells (Fig. 4c). Therefore, when working with armored CAR T-cells with engineered cytokine signaling, the identification of the specific cytokine overexpression represents a useful strategy to identify activated CAR T-cells. To test this approach, we evaluated IL-18 immunolabeling as a means to identify activated human CAR T-cells armored with human IL-18 cytokine expression. IL-18 immunolabeling was observed in limited numbers of CAR T-cells in the inflammatory infiltrates (Fig. 4d).

Finally, using multiplexed ISH, the UTRs probe can be combined with the specific CAR binding target, enabling a precise mapping of the spatial relationships between CAR T-cells and target-expressing cells (Fig. 4e).

## Discussion

The validation of reliable markers distinguishing “human” from “mouse” cells in chimeric tissues is critical due to the evolving complexity of chimeric mouse models that incorporate an increasing variety of human components. This study evaluated 3 broad-spectrum human-specific antibodies with the goal of identifying ideal markers for the detection of human cells in chimeric mice. Targets of broad-spectrum antibodies aimed at differentiating species of origin should be expressed in virtually all cells and tissue types, producing a strong, easily identifiable chromogenic signal. Importantly, while our TMA approach enabled screening across a wide range of normal and neoplastic tissues, it provided only a limited number of observations for many cell types (eg, most epithelial cell types, reproductive tissues, and central nervous system tissues). Many cell populations had just one biological replicate and some (eg, ovary) were not available for evaluation, which restricts the broad relevance of some of the conclusions drawn from this experiment.

HLA-A is one of the most widely used and effective antibodies for identifying human-derived cells in mouse tissues, and its strong membranous labeling provides a clear and easily interpreted signal. HLA-A is present on virtually all nucleated cells in human tissues, with the exception of neurons and germ cells, and is expressed only in human-derived cells in chimeric mice. However, HLA-A is expressed at low levels in some normal tissues, such as the renal tubules,^22,48,61^ and its expression may have been below detectable limits in the TMA. HLA-A is weakly expressed on skeletal muscle tissue in the absence of inflammation and is variably expressed in rhabdomyosarcomas.^16,56^ Pathologists seeking to visualize the invasive tumor front in a xenograft model of rhabdomyosarcoma may be better served by selecting a different marker richly expressed in skeletal muscle tissue, such as hMito. Furthermore, loss or downregulation of HLA-A expression as a mechanism of immune escape has been reported in a number of naturally occurring neoplasms, including colorectal, prostate, breast, and lung cancers, among others.^3,5,21,34,62^ As noted in this study, HLA-A was strongly expressed in the normal pulmonary epithelium core but not in the pulmonary adenocarcinoma sample.^7,20^ In breast cancer and melanoma, metastatic lesions are associated with HLA-A loss, which may complicate cell identification in mice receiving metastatic patient-derived xenografts from these and other tumors.^29,49,64^ As noted, off-target cytoplasmic HLA-A labeling was identified in rare mast cells; nonspecific mast cell labeling is common in protocols using immunoperoxidase techniques for IHC.^8,52^

Ku80 was found to produce clean, strong nuclear labeling in both the TMA and in human immune cells in humanized mice. Compared with the membranous labeling of HLA-A, the exclusive nuclear distribution of Ku80 created a sparse pattern with less obvious demarcation between neighboring human and mouse cells in chimeric samples. On the other hand, its exclusive nuclear labeling could be conveniently leveraged for multiplexing with membranous or cytoplasmic markers for which there are not human-specific antibodies available. In general, Ku80 provided complementary labeling to HLA-A, with only sperm cells being negative for both markers. Critically, Ku80 provided coverage where HLA-A expression was lacking. Ku80 was strongly expressed in breast duct epithelium, renal tubular epithelium, and thyroid epithelium and moderately expressed in smooth muscle and cerebral neurons, which were all negative for HLA-A.^9,24,25,31^ Faint nuclear labeling of some normal mouse tissues (renal tubules, testis, and adrenal gland) with human Ku80 was interpreted as low-affinity binding of the homologous target in mice.

The hMito marker was found to have limited utility. Mitochondria are widely distributed through most tissues, and no cell populations were considered to be completely negative on the TMA.^17^ However, the pattern of fine, punctate cytoplasmic signal resulted in the appearance of weak labeling in tissues with lower mitochondrial densities, which complicated the clear identification of human cells in chimeric tissues. It is plausible that this marker may be more useful when evaluated using highly sensitive digital methods that may be better suited to detecting the weaker signal in some cell types. Despite this shortcoming, hMito performed well in neoplastic tissues and was specific for human cells with no off-target labeling in NSG mouse tissues, indicating that it may be a suitable choice for the identification of human tissues (especially tumors) with high mitochondrial content (eg, rhabdomyosarcoma or lymphoma).

Crucially, no single cell type was negative for all 3 markers, proving that immunohistochemical markers are available for virtually every human cell type engrafted in chimeric mice.

Engrafting human tissues in mice may occasionally give rise to unexpected pathologies, such as aberrant proliferation of human B cells driven by the Epstein-Barr virus in patient-derived xenograft models.^38,47,58^ With this in mind, special focus was given to the performance of the 3 tested antibodies on both normal and neoplastic lymphocytes. HLA-A produced strong labeling on healthy lymphocytes and in both of the lymphoid neoplasms included on the TMA, but Ku80 and hMito expression was weak to absent in some healthy lymphocyte populations. However, both Ku80 and hMito produced strong signal in the diffuse large B cell lymphoma and the Hodgkin lymphoma, which in the case of hMito may be attributable to increased mitochondrial activity in lymphoma and presents a unique use for an otherwise limited marker.^50,51^

The narrow-spectrum antibodies included in this study were mainly tested to assess their species-specificity on FFPE tissues from humanized mice to enable accurate mapping of spatial relationships among different human and murine cell subsets. We provided several examples where the application of human- and mouse-specific immunolabeling methods is pivotal to diagnosing post-transplant disorders, as well as spontaneous conditions commonly affecting chimeric mice.^1,28,57,60^ Spatial and morphological definition of different chimeric tissue components is also critical to complement data obtained using tissue-destructive approaches such as multiplex flow cytometry.^23^ Historically, flow cytometry has been the preferred method for determining the extent and nature of chimerism in various tissues from chimeric mouse models.^26,27,37^ While it allows for quantitative and qualitative analysis of complex cell populations, flow cytometry fails to capture any topographic information regarding actual cell distribution and interaction within the tissue itself.^23^ This has led to misleading interpretations regarding the nature of chimerism in diverse mouse models.^43,57,65^

The development of adoptive immunotherapy products such as CAR T-cells relies on efficacy and/or toxicity testing in severely immunodeficient mice carrying human tumor xenografts expressing the target of interest. Investigating the spatial distribution of human CAR T-cells in tissue samples from treated mice is critical to the evaluation of important experimental readouts like evidence of tumor cell targeting, on-target/ off-tumor cytotoxicity, and long-term persistence of CAR T-cells. The use of broad-spectrum human-specific leukocyte or T-cell markers (eg, CD45 LCA or CD3Ɛ) might be sufficient for general identification of human T-cells in tissues from treated mice, but this approach may be inadequate if the main goal is distinguishing between human T-cells with and without successful CAR transduction. The identification of the actual fraction of successfully transduced CAR T-cells included in the adoptive T-cell transfer provides critical insight for interpreting tissue changes potentially associated with cytotoxicity. CAR expression can be confirmed using antibodies targeting unmodified structural components, like the linker sequence of single-chain variable fragments. Unfortunately, these antibodies have not been established for immunolabeling in FFPE tissue sections.^59^ The use of ISH with probes targeting invariant elements of the viral vectors (eg, UTRs) for CAR transduction avoids this shortcoming. However, as demonstrated in our work, these targets may be shared by different viral vectors within the same family, potentially compromising the specificity of this approach. This issue becomes particularly relevant when similar viral vectors are used for both CAR transduction and other applications within the same study. To address the problem of target overlap, recent studies have successfully utilized ISH probes that bind to CAR-specific RNA transcripts, such as those encoding the spacer or hinge regions, instead of targeting viral vector-specific sequences.^14^

Species-specific antibodies for many mouse and human targets are widely commercially available, but cross-reactivity may not be well-described in published literature or fully tested by the vendor.^4^ This creates a source of frustration when identifying quality antibodies that can be used effectively for the immunophenotyping lesions in chimeric mice.^4^ In this context, our work provides a useful reference for selecting commercially available human-specific or mouse-specific antibodies targeting the main cell populations of hematopoietic origin. The application of a rigorous validation process, featuring the parallel testing of relevant human, murine, and humanized mouse tissue samples, allowed the identification of matching human-specific and mouse-specific markers for key myeloid and lymphoid cell populations. Nevertheless, species-specific markers could not be validated for some of the key targets, including, among others, CD11b, CD204, and FoxP3. When dealing with these cross-binding antibodies recognizing both the human and the mouse homologs, multiplex IF combining human-specific and mouse-specific broad-spectrum markers represented a valid alternative to study the origin of target expression in chimeric tissues.

In conclusion, understanding target selection, ideal applications, and limitations to specific labeling choices is essential for pathologists evaluating tissues from chimeric mice. The methods presented here illustrate the validation and application of various labeling strategies and protocols to differentiate between specific mouse and human components in FFPE samples from humanized mice. These approaches frequently complement each other, underscoring the importance of developing an arsenal of multiple markers, both broad- and narrow-spectrum, for the diverse cell phenotyping needs of chimeric mice.^32,35,41,45,63^ Although not comprehensive, this work provides a valuable experimental and diagnostic reference for pathologists and investigators working with humanized models and seeking to add spatial resolution to the complex landscape of chimeric tissues.

## Supplemental Material

sj-pdf-1-vet-10.1177_03009858251386916 – Supplemental material for Techniques to study chimerism at the tissue level in humanized mice Supplemental material, sj-pdf-1-vet-10.1177_03009858251386916 for Techniques to study chimerism at the tissue level in humanized mice by Arin Cox, Esha Banerjee, Jillian Verrelle, Elinor Willis, Charles-Antoine Assenmacher, Giovanni Finesso, James C. Tarrant and Enrico Radaelli in Veterinary Pathology
