# Supplementary material for: Techniques to study chimerism at the tissue level in humanized mice
Source: Vet Pathol. 2025 Nov 21;63(3):478–89. doi: 10.1177/03009858251386916 (PMC13036268; doi:10.1177/03009858251386916)
Supplement: sj-pdf-1-vet-10.1177_03009858251386916 – Supplemental material for Techniques to study chimerism at the tissue level in humanized mice [file sj-pdf-1-vet-10.1177_03009858251386916.pdf]

Supplemental Materials

Techniques to study chimerism at the tissue level in humanized mice

Arin Cox, Esha Banerjee, Jill Verrelle, Elinor Willis, Charles-Antoine Assenmacher,

Giovanni Finesso, James Carmine Tarrant, Enrico Radaelli

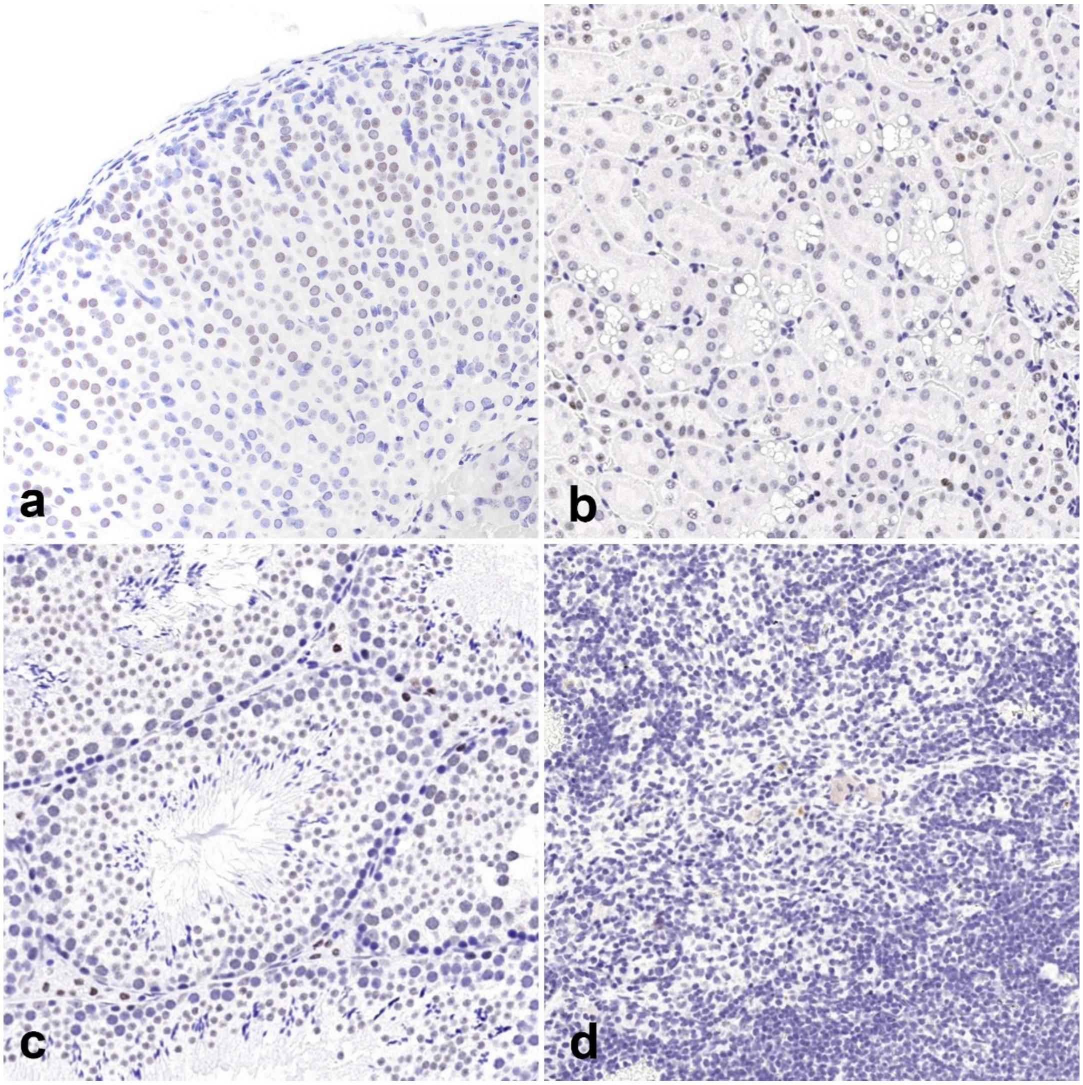

Supplemental Figure S1. Nonspecific and low-affinity labeling of broad-spectrum human antibodies in normal adult C57BL/6 mice. (a-c) There is weak, low-affinity labeling for Ku80 in the (a) adrenal cortex, (b) renal tubules, and (c) testes. (d) There is weak, nonspecific cytoplasmic labeling of HLA-A in scattered mononuclear cells (likely mast cells) in the lymph node, as well as faint golden-brown granules of endogenous pigment.

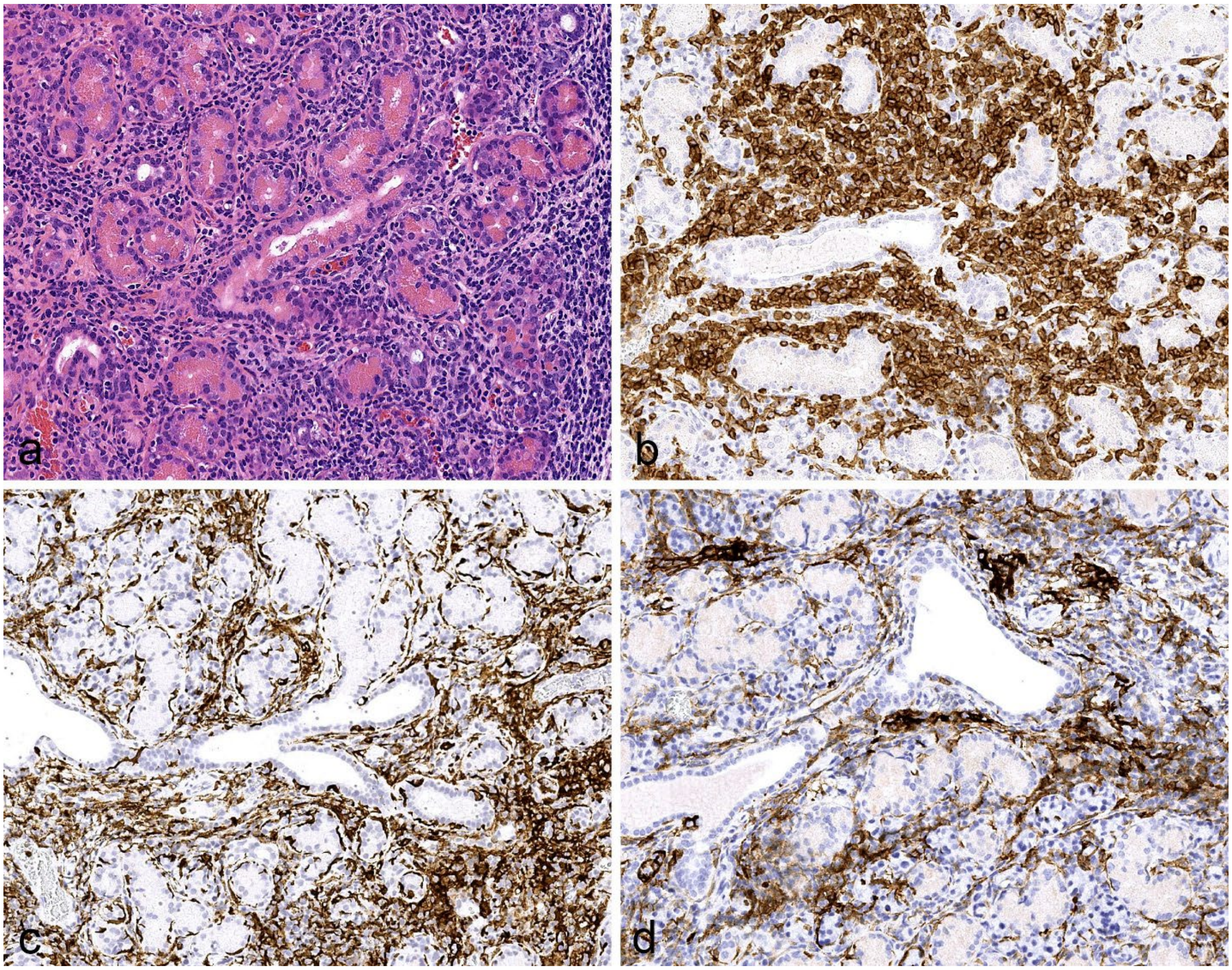

Supplemental Figure S2. Lesion compatible with xenogeneic graft versus host disease in the submandibular salivary gland of an NSG mouse treated with human CAR T-cells. (a) The interstitium of the gland is expanded by diffuse lymphohistiocytic infiltrates associated with fibrosis and acinar loss. Hematoxylin and eosin. (b) One of the main components of the infiltrate consists of human CAR T-cells, as demonstrated by the diffuse expression of the human-specific CD45 LCA marker. Human-specific CD45 LCA immunohistochemistry. (c). F4/80-positive mouse macrophages represent a considerable fraction of the infiltrate. Mouse-specific F4/80 immunohistochemistry. (d) In addition, many murine macrophages and possibly dendritic cells act as antigen-presenting cells by expressing the costimulatory molecule CD86. Mouse-specific CD86 immunohistochemistry.

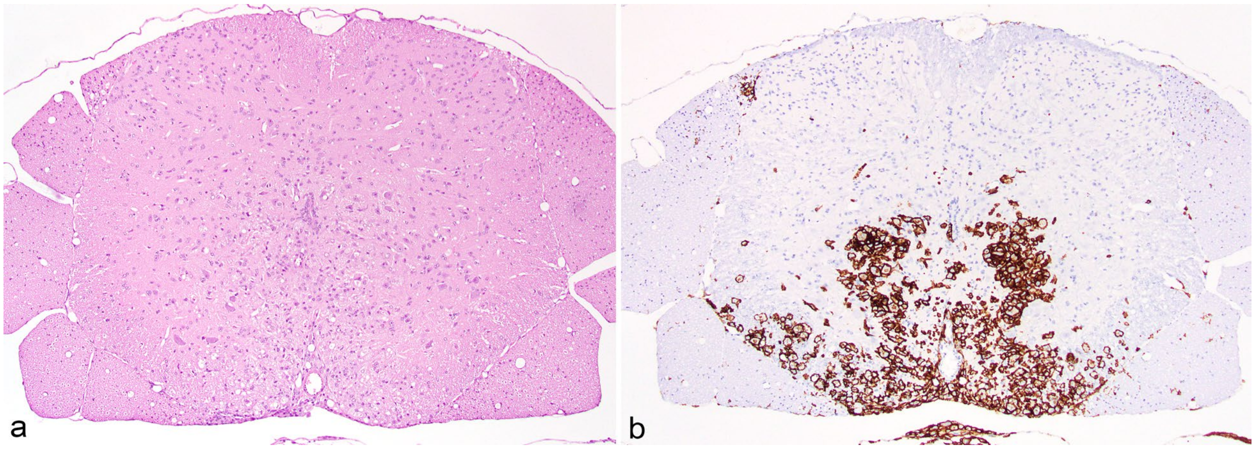

Supplemental Figure S3. Spinal cord. Chimeric myeloid cell hyperactivation syndrome in a NOG-EXL mouse humanized with human CD34+ hematopoietic stem cells. Clinically, the mouse was euthanized 26 weeks after humanization upon developing paraparesis. (a) The ventral funiculi, the ventral portion of the fasciculus proprius, and the ventral white commissure of the lumbar spinal cord are hypercellular with degenerative changes consisting of axonal damage/loss or degeneration. Hematoxylin and eosin. (b) The affected regions are heavily infiltrated by CD33-positive human macrophages. Human-specific CD33 immunohistochemistry.

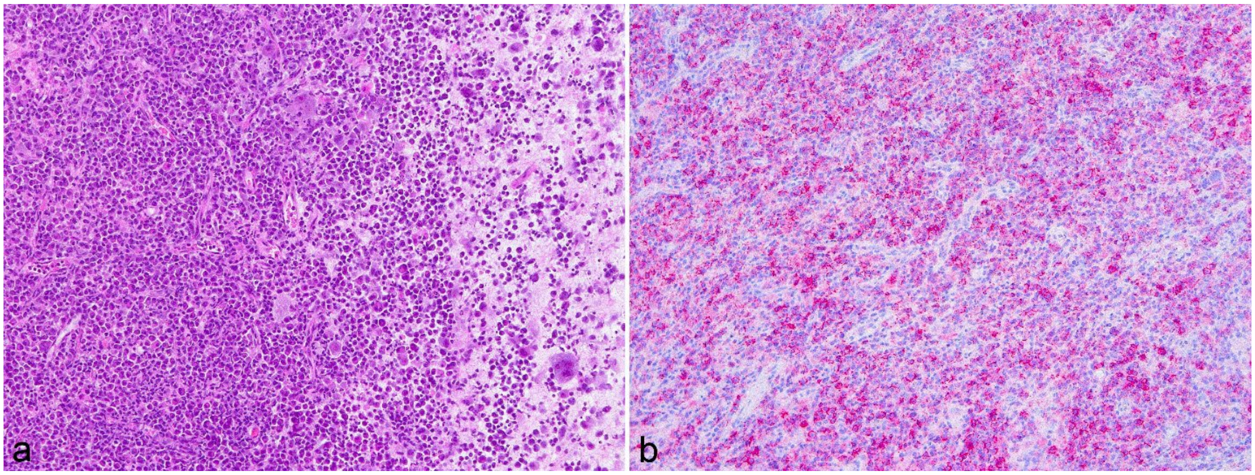

Supplemental Figure S4. Post-transplant lymphoproliferative disorder in a NOG mouse with a metastatic melanoma patient-derived xenograft (PDX). (a) The subcutis and fascia at the PDX transplantation site are densely infiltrated by atypical round cells with plasmacytoid morphology consistent with a B-cell lymphoma with plasma cell differentiation. Hematoxylin and eosin. (b) The diffuse expression of the human-specific CD138 marker confirms the plasma cell differentiation and the human origin of the lymphoma. Human-specific CD138 immunohistochemistry.

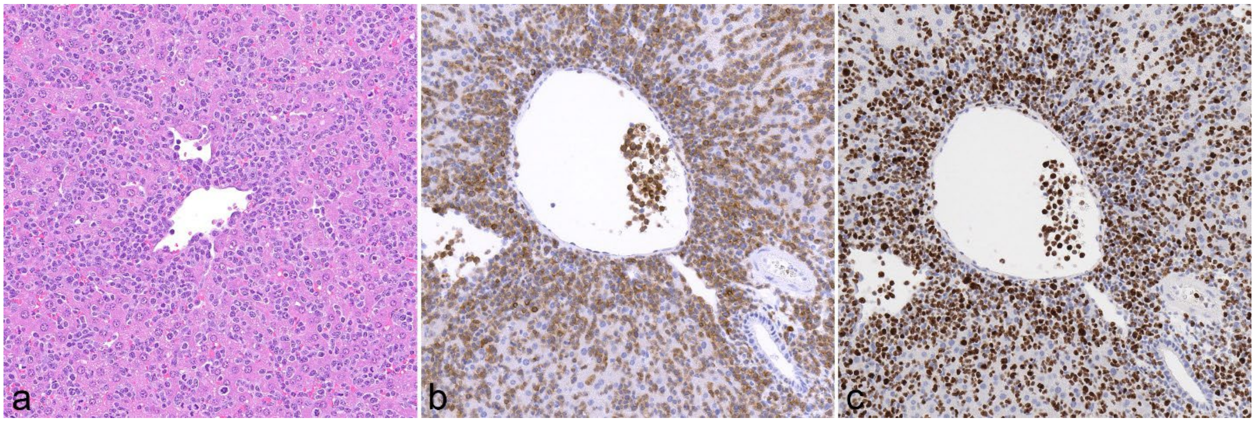

Supplemental Figure S5. Aberrant human CAR T-cell proliferation in a treated NSG mouse. (a) The liver is severely infiltrated by atypical lymphoid cells dissecting through the hepatic cords. Hematoxylin and eosin. (b) The infiltrating lymphoid cells diffusely express human CD3, confirming the human T-cell origin. Human-specific CD3 immunohistochemistry. (c) The same population of atypical human T-cells exhibits an extremely high proliferative index, as demonstrated by the diffuse Ki-67 nuclear expression. Ki-67 immunohistochemistry.

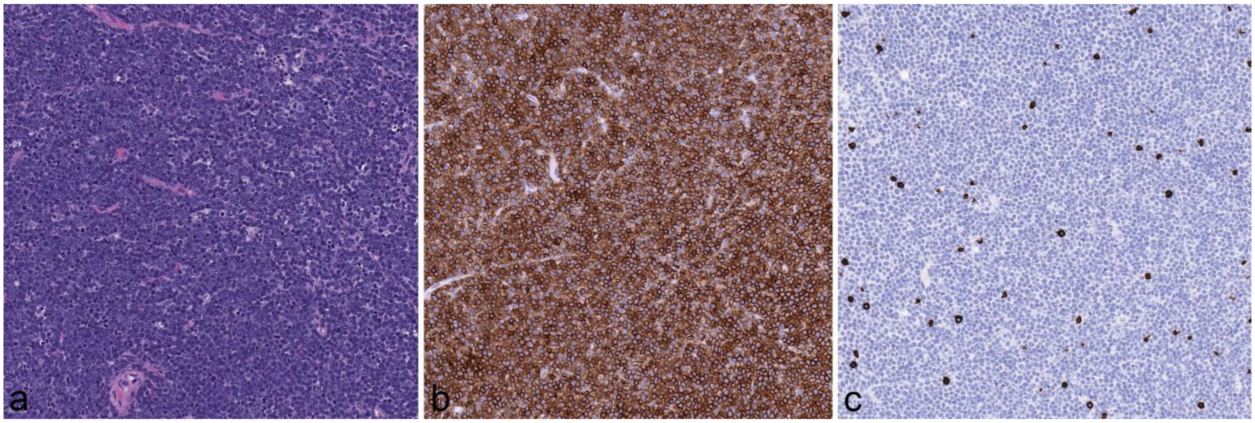

Supplemental Figure S6. Spontaneous mouse T-cell lymphoma in an NSG mouse treated with CAR T-cells. At necropsy, the mouse presented with splenomegaly, mesenteric lymphadenomegaly, and a large white mass infiltrating the cranial and mid portion of the thoracic cavity. (a) The thoracic mass comprises a monomorphic population of mid-sized lymphoid cells with scant cytoplasm and a high mitotic index compatible with a lymphoma diagnosis. Hematoxylin and eosin. (b) This atypical population of lymphoid cells is diffusely positive for mouse-specific CD45 LCA, confirming the mouse origin of the neoplasm. Mouse CD45 LCA immunohistochemistry. (c) Scattered human CD45 LCA-positive lymphocytes, presumably CAR T-cells, are also evident within the tumor. Human CD45 LCA immunohistochemistry.

Supplemental Table S1. Comprehensive list of immunohistochemical markers specific for mouse or human tissues.

Epitope/Target	Species Reactivity	Primary Antibody	Primary Use	Tissue(s) Used for Validation	Working Dilution
Arginase-1	Human and Mouse	Arginase-1, CST, #93668, Rb mAb.	M2 macrophages	Brain from a C57BL/6J mouse infected with *T. gondii*; Human tonsil with chronic inflammation; Spleen and liver from a humanized NSG mouse with chimeric MCH syndrome	1/300(IHC); 1/600 (IF)
ASC/PYCARD	Mouse	ASC/TMS1, CST, #67824, Rb mAb	Pyroptosis	Ear pinna from a C57BL/6NCr mouse experimentally infected with Leishmania major; Human tonsil with chronic inflammation	1/300
ASC/PYCARD	Human	ASC/TMS1, CST, #13833, Rb mAb	Pyroptosis	Ear pinna from a C57BL/6NCr mouse experimentally infected with Leishmania major; Human tonsil with chronic inflammation	1/4000
CD3	Human and Mouse	CD3ε, Bio-Rad, MCA1477T, Rat mAb	T cells	Lymphoid tissues from a C57BL/6J mouse; Human lymph node and tonsil with chronic inflammation; Lungs and liver from a NSG mouse with aberrant proliferation of human CAR T cells	1/600(IHC); 1/1200 (IF)
CD3	Mouse and Human	CD3ε, CST, #99940, Rb mAb	T cells	Lymphoid tissues from a C57BL/6J mouse; Human lymph node and tonsil with chronic inflammation; Lungs and liver from a NSG mouse with aberrant proliferation of human CAR T cells	1/100
CD3	Human	CD3, Agilent (DAKO), M7254, Ms mAb	T cells	Lymphoid tissues from a C57BL/6J mouse; Human lymph node and tonsil with chronic inflammation; Lungs and liver from a NSG mouse with aberrant proliferation of human CAR T cells	1/150
CD4	Human	CD4 SP35, Cell Marque, 104R-14, Rb mAb	Helper T cells	Lymphoid tissues from a C57BL/6J mouse; Human lymph node and tonsil with chronic inflammation; Lungs and liver from a NSG mouse with aberrant proliferation of human CAR T cells	1/50(IHC); 1/1500 (IF)
CD4	Mouse	CD4, Abcam, ab183685, Rb mAb	Helper T cells	Lymphoid tissues from a C57BL/6J mouse; Human lymph node and tonsil with chronic inflammation; Lungs and liver from a NSG mouse with aberrant proliferation of human CAR T cells	1/2000(IHC); 1/1000 (IF)
CD8	Human	CD8α, CST, #85336, Rb mAb	Cytotoxic T cells	Lymphoid tissues from a C57BL/6J mouse; Human lymph node and tonsil with chronic inflammation; Lungs and liver from a NSG mouse with aberrant proliferation of human CAR T cells	1/300(IHC); 1/900 (IF)
CD8	Mouse	CD8α, CST, #98941, Rb mAb	Cytotoxic T cells	Lymphoid tissues from a C57BL/6J mouse; Human lymph node and tonsil with chronic inflammation; Lungs and liver from a NSG mouse with aberrant proliferation of human CAR T cells	1/1000(IHC); 1/1500 (IF)
CD11b	Human and Mouse	CD11b, CST, 93169, Rb mAb	Macrophages and granulocytes	Lymphoid tissues from a C57BL/6J mouse; Human tonsil with chronic inflammation; Spleen and liver from a humanized NSG mouse with chimeric MCH syndrome	1/500 (IHC); 1/2000 (IF)
CD11c	Mouse	CD11c, CST, #97585, Rb mAb	Myeloid-derived DCs	Lymphoid tissues from a C57BL/6J mouse; Human tonsil with chronic inflammation; Skin, lungs, and salivary glands from a human CAR T cell-treated NSG mouse with xGvHD	1/150(IHC); 1/500(IF)
CD11c	Human and Mouse	CD11c, CST, #45581, Rb mAb	Myeloid-derived DCs	Lymphoid tissues from a C57BL/6J mouse; Human tonsil with chronic inflammation; Spleen and liver from a humanized NSG mouse with chimeric MCH syndrome	1/1000 (IHC); 1/2000 (IF)
CD20	Mouse	CD20, CST, #70168, Rb mAb	B cells	Lymphoid tissues from a C57BL/6J mouse; Human tonsil with chronic inflammation; Metastatic melanoma PDX from a NOG mouse with EBV-associated PTLD	1/300
CD20	Human	CD20, Thermo Scientific, RB9013P, Rb pAb	B cells	Lymphoid tissues from a C57BL/6J mouse; Human tonsil with chronic inflammation; Metastatic melanoma PDX from a NOG mouse with EBV-associated PTLD	1/500
CD20	Human	CD20, Agilent (DAKO), M0755, L26, Ms mAb	B cells	Lymphoid tissues from a Crl:CD1(ICR) mouse; Human tonsil with chronic inflammation; Metastatic melanoma PDX from a NOG mouse with EBV-associated PTLD	1/100
CD31	Mouse	CD31, Dianova, DIA-310, Rat mAb	Endothelial cells	Lymphoid tissues from a C57BL/6J mouse; Human tonsil with chronic inflammation	1/100
CD31	Mouse	CD31, CST, #77699, Rb mAb	Endothelial cells	Lymphoid tissues from a C57BL/6J mouse; Human tonsil with chronic inflammation	1/300
CD31	Human	CD31, Abcam, ab28364, Rb pAb	Endothelial cells	Lymphoid tissues from a C57BL/6J mouse; Human tonsil with chronic inflammation	1/100
CD31	Human	CD31, Agilent (DAKO), M0823, Ms mAb	Endothelial cells	Lymphoid tissues from a C57BL/6J mouse; Human tonsil with chronic inflammation	1/20
CD33/Siglec-3	Human	CD33, Abcam, ab199432, Rb mAb	Macrophages and granulocytes	Lymphoid tissues from a C57BL/6J mouse; Human tonsil with chronic inflammation; Spleen and liver from a humanized NSG mouse with chimeric MCH syndrome	1/200(IHC); 1/1500 (IF)
CD40	Mouse	CD40, CST, #86165, Rb mAb	Antigen-presenting cells	Lymphoid tissues from a C57BL/6J mouse; Human lymph node and tonsil with chronic inflammation; Spleen and liver from a humanized NSG mouse with chimeric MCH syndrome	1/150
CD40	Human	CD40, CST, 40868, Rb mAb	Antigen-presenting cells	Lymphoid tissues from a C57BL/6J mouse; Human lymph node and tonsil with chronic inflammation; Spleen and liver from a humanized NSG mouse with chimeric MCH syndrome	1/200(IHC); 1/600 (IF)
CD45 LCA	Mouse	CD45-LCA, BD Biosciences, 553076, Rat mAb.	All leukocytes	Lymphoid tissues from a C57BL/6J mouse; Human tonsil with chronic inflammation; Spleen and liver from a humanized NSG mouse with chimeric MCH syndrome	1/300(IHC); 1/1000 (IF)
CD45 LCA	Mouse	CD45-LCA, CST, #13917, Rb mAb	All leukocytes	Lymphoid tissues from a C57BL/6J mouse; Human tonsil with chronic inflammation; Spleen and liver from a humanized NSG mouse with chimeric MCH syndrome	1/300(IHC); 1/1000 (IF)
CD45 LCA	Human	CD45-LCA, CST, #70257, Rb mAb	All leukocytes	Lymphoid tissues from a C57BL/6J mouse; Human tonsil with chronic inflammation; Spleen and liver from a humanized NSG mouse with chimeric MCH syndrome	1/400(IHC); 1/1200 (IF)
CD45 B220	Mouse	CD45R/B220, BD Biosciences, 550286, Rat mAb	B cells	Lymphoid tissues from a C57BL/6J mouse; Human tonsil with chronic inflammation; Metastatic melanoma PDX from a NOG mouse with EBV-associated PTLD	1/500(IHC); 1/1000 (IF)
CD45RO	Human	CD45RO, CST, #55618, Ms mAb.	Memory T cells	Lymphoid tissues from a C57BL/6J mouse; Human tonsil with chronic inflammation; Lungs and liver from a NSG mouse with aberrant proliferation of human CAR T cells	1/200
CD56	Human	CD56/NCAM1, CST, 99746, Rb mAb	NK cells	Lymphoid tissues from a C57BL/6J mouse; Human tonsil with chronic inflammation; Skin, lungs, and salivary glands from a melanoma PDX-engrafted NOG mouse with xGvHD	1/200
CD68	Human	CD68, Leica/Novocastra, NCL-L-CD68 (CD68-L-CE), Ms mAb	Macrophages	Lymphoid tissues from a C57BL/6J mouse; Human tonsil with chronic inflammation; Spleen and liver from a humanized NSG mouse with chimeric MCH syndrome	1/150
CD68	Mouse	CD68, CST, #97778, Rb mAb	Macrophages	Lymphoid tissues from a C57BL/6J mouse; Human tonsil with chronic inflammation; Spleen and liver from a humanized NSG mouse with chimeric MCH syndrome	1/500(IHC); 1/1500 (IF)
CD79	Human and Mouse	CD79b, CST, #96024, Rb mAb	B cells	Lymphoid tissues from a C57BL/6J mouse; Human tonsil with chronic inflammation; Metastatic melanoma PDX from a NOG mouse with EBV-associated PTLD	1/300(IHC); 1/1000 (IF)
CD86	Mouse	CD86, CST, #19589, Rb mAb	T cell activation by APCs	Lymphoid tissues from a C57BL/6J mouse; Human lymph node and tonsil with chronic inflammation; Spleen and liver from a humanized NSG mouse with chimeric MCH syndrome	1/300
CD86	Human	CD86, CST, #91882, Rb mAb	T cell activation by APCs	Lymphoid tissues from a C57BL/6J mouse; Human lymph node and tonsil with chronic inflammation; Spleen and liver from a humanized NSG mouse with chimeric MCH syndrome	1/200
CD117/Kit	Mouse	cKit, abcam, ab231780, Rb mAb	Mast cells	Mouse GIST; Human GIST; Pancreas and liver from a humanized NSG mouse with chimeric MCH syndrome	1/200
CD117/Kit	Human	C-Kit, CST, #37805, Rb mAb	Mast cells	Mouse GIST; Human GIST; Pancreas and liver from a humanized NSG mouse with chimeric MCH syndrome	1/300(IHC); 1/1000 (IF)
CD117/Kit	Human and Mouse	C-Kit/CD117, Agilent (DAKO), A4502, Rb pAb	Mast cells	Mouse GIST; Human GIST; Pancreas and liver from a humanized NSG mouse with chimeric MCH syndrome	1/50

CD138	Human	CD138, Agilent (DAKO), IR642, MI15, Ms mAb	Plasma cells	Lymphoid tissues from a Crl:CD1(ICR) mouse; Human tonsil with chronic inflammation; Metastatic melanoma PDX from a NOG mouse with EBV-associated PTLD	1/100 (IHC); 1/200 (IF)
CD138	Mouse	CD138, BD Biosciences, 553712, Rat mAb	Plasma cells	Lymphoid tissues from a C57BL/6J mouse; Human tonsil with chronic inflammation; Metastatic melanoma PDX from a NOG mouse with EBV-associated PTLD	1/1000
CD204	Human and Mouse	CD204, TransGenic Inc, KT022, Ms mAb	Macrophages	Lymphoid tissues from a C57BL/6J mouse; Human tonsil with chronic inflammation; Spleen and liver from a humanized NSG mouse with chimeric MCH syndrome	1/1500 (IHC); 1/5000 (IF)
CD206/MRC1	Human and Mouse	CD206/MRC1 (E6T5J), CST, #24595, Rb mAb.	M2 macrophages	Brain from a C57BL/6J mouse infected with T. gondii; Human tonsil with chronic inflammation; Spleen and liver from a humanized NSG mouse with chimeric MCH syndrome	1/300 (IHC); 1/1000 (IF)
F4/80	Mouse	F4/80, CST, #70076, Rb mAb	Macrophages	Lymphoid tissues from a Crl:CD1(ICR) mouse; Human tonsil with chronic inflammation; Spleen and liver from a humanized NSG mouse with chimeric MCH syndrome	1/1000(IHC); 1/3000 (IF)
FoxP3	Human and Mouse	FoxP3, CST, #12653, Rb mAb	Regulatory T cells	Lymphoid tissues from a C57BL/6J mouse; Human tonsil with chronic inflammation; Skin, lungs, and salivary glands from a melanoma PDX-engrafted NOG mouse with xGvHD	1/300(IHC); 1/1000 (IF)
Granzyme B	Human and Mouse	Granzyme B, Abcam, ab4059, Rb pAb.	Cytotoxic T cells, NK cells, mast cells	Lymphoid tissues from a C57BL/6J mouse; Human tonsil with chronic inflammation; Skin, lungs, and salivary glands from a melanoma PDX-engrafted NOG mouse with xGvHD	1/200(IHC); 1/1000 (IF)
Granzyme B	Mouse	Granzyme B, CST, #44153, Rb mAb.	Cytotoxic T cells, NK cells, mast cells	Lymphoid tissues from a C57BL/6J mouse; Human tonsil with chronic inflammation; Skin, lungs, and salivary glands from a melanoma PDX-engrafted NOG mouse with xGvHD	1/200(IHC); 1/800 (IF)
IBA1	Human and Mouse	IBA1, WAKO, 019-19741, Rb pAb.	Macrophages and dendritic cells	Lymphoid tissues from a C57BL/6J mouse; Human tonsil with chronic inflammation; Spleen and liver from a humanized NSG mouse with chimeric MCH syndrome	1/1500(IHC); 1/3000 (IF)
iNOS	Mouse	iNOS, Abcam, ab15323, Rb pAb.	M1 macrophages	Lymphoid tissues from a C57BL/6J mouse; Human tonsil with chronic inflammation; Spleen and liver from a humanized NSG mouse with chimeric MCH syndrome	1/100 (IHC); 1/500 (IF)
iNOS	Human and Mouse	iNOS, Novus Bio, NB300-605, Rb pAb.	M1 macrophages	Brain from a C57BL/6J mouse infected with T. gondii; Human tonsil with chronic inflammation; Spleen and liver from a humanized NSG mouse with chimeric MCH syndrome	1/800(IHC); 1/2000 (IF)
IRF4/MUM1	Human and Mouse	IRF-4, CST, #62834, Rb mAb.	Plasma cells	Lymphoid tissues from a C57BL/6J mouse; Human tonsil with chronic inflammation; Metastatic melanoma PDX from a NOG mouse with EBV-associated PTLD	1/2500
Ly-6G	Mouse	Ly-6G, CST, #87048, Rb pAb.	Neutrophils	Lymphoid tissues from a C57BL/6J mouse; Human tonsil with chronic inflammation; Spleen and liver from a humanized NSG mouse with chimeric MCH syndrome	1/100
MDC/CCL22	Mouse	MDC (EPR1362), Abcam, ab124768, Rb mAb.	Activated macrophages and dendritic cells	Lymphoid tissues from a C57BL/6J mouse; Human tonsil with chronic inflammation; Spleen and liver from a humanized NSG mouse with chimeric MCH syndrome	1/300
MHC class II	Human	MHC Class II (HLA-DR), Agilent (DAKO), m0746, Ms mAb.	Antigen-presenting cells	Lymphoid tissues from a C57BL/6J mouse; Human tonsil with chronic inflammation; Spleen and liver from a humanized NSG mouse with chimeric MCH syndrome	1/200(IHC); 1/600 (IF)
MHC class II[a]	Mouse	MHC II, SouthernBiotech, 1895-01, Rt mAb.	Antigen-presenting cells	Lymphoid tissues from a C57BL/6J mouse; Human tonsil with chronic inflammation	1/20 (IHC)
MHC class II[a]	Mouse	MHC Class II, Bio-Legned, 107601, Rat mAb.	Antigen-presenting cells	Lymphoid tissues from a C57BL/6J mouse; Human tonsil with chronic inflammation	1/200(IHC); 1/600 (IF)
MPO	Human and Mouse	MPO, Agilent (DAKO), A0398, Rb pAb.	Neutrophils and myeloid precursors	Bone marrow from a C57BL/6J mouse; Human bone marrow; Spleen and liver from a humanized NSG mouse with chimeric MCH syndrome	1/1500
NK1.1[a]	Mouse	NK1.1/CD161, CST, #39197, Rb mAb.	Natural killer cells	Lymphoid tissues from a C57BL/6J mouse; Human tonsil with chronic inflammation	1/500
TfR1	Human and Mouse	Transferrin Receptor CD71, Abcam, ab214039, Rb mAb.	Erythroid precursors	Bone marrow from a C57BL/6J mouse; Human bone marrow	1/1000(IHC); 1/3000 (IF)

[a] Recgonized target not expressed in NOD mice

Abbreviations: CST, Cell Signaling Technology; Rb, rabbit; mAb, monoclonal antibody; MCH, myeloid cell hyperactivation; DCs, dendritic cells; NK, natural killer; PDX, patient derived xenografts; EBV, Epstein-Barr Virus; PTLD, post-transplant lymphoproliferative disorder; GIST, gastrointestinal stromal tumor; APCs, antigen presenting cells; xGvHD, xenogeneic graft-

Supplemental Table S2. Intensity scoring of HLA-A, Ku80, and hMito in cell populations human TMA cores, with basic statistics.

			hHLA-A		*Median*		*IQR*		hKu80		*Median*		*IQR*		hMito		*Median*		*IQR*	
**Cell Type**	**# Normal**	**# Neoplastic**	**Normal**	**Neoplastic**	**Normal**	**Neoplastic**	**Normal**	**Neoplastic**	**Normal**	**Neoplastic**	**Normal**	**Neoplastic**	**Normal**	**Neoplastic**	**Normal**	**Neoplastic**	**Normal**	**Neoplastic**	**Normal**	**Neoplastic**
Squamous epithelium	2[a]	4	3	3	n/a	3	n/a	0	3	3	n/a	3	n/a	0	3	3	n/a	2.75	n/a	0.5
Gastric mucosa	2	1	3	3	n/a	n/a	n/a	n/a	3	3	n/a	n/a	n/a	n/a	3	3	n/a	n/a	n/a	n/a
Small intestine mucosa	1	0	3	n/a	n/a	n/a	n/a	n/a	3	n/a	n/a	n/a	n/a	n/a	3	n/a	n/a	n/a	n/a	n/a
Large intestine mucosa	3	3	3	3	3	2.67	0	1	3	3	3	3	0	0	3	3	2.67	3	1	0
Salivary duct epithelium	1	0	2	n/a	n/a	n/a	n/a	n/a	3	n/a	n/a	n/a	n/a	n/a	3	n/a	n/a	n/a	n/a	n/a
Breast duct epithelium	1	1	0	0	n/a	n/a	n/a	n/a	3	3	n/a	n/a	n/a	n/a	3	3	n/a	n/a	n/a	n/a
Renal tubular epithelium	2	1	0	3	n/a	n/a	n/a	n/a	3	3	n/a	n/a	n/a	n/a	3	3	n/a	n/a	n/a	n/a
Urothelium	1	0	2	n/a	n/a	n/a	n/a	n/a	3	n/a	n/a	n/a	n/a	n/a	2	n/a	n/a	n/a	n/a	n/a
Thyroid epithelium	1	1	1	3	n/a	n/a	n/a	n/a	3	3	n/a	n/a	n/a	n/a	1	3	n/a	n/a	n/a	n/a
Pulmonary epithelium	1	1	3	0	n/a	n/a	n/a	n/a	3	3	n/a	n/a	n/a	n/a	1	2	n/a	n/a	n/a	n/a
Gallbladder epithelium	1	1	3	3	n/a	n/a	n/a	n/a	3	3	n/a	n/a	n/a	n/a	3	3	n/a	n/a	n/a	n/a
Hepatocytes	1	1	3	2	n/a	n/a	n/a	n/a	2	3	n/a	n/a	n/a	n/a	3	3	n/a	n/a	n/a	n/a
Adrenal cortex	1	0	3	n/a	n/a	n/a	n/a	n/a	3	n/a	n/a	n/a	n/a	n/a	3	n/a	n/a	n/a	n/a	n/a
Lymphocytes	8	2	3	3	2.875	n/a	0.5	n/a	3	3	3	n/a	0	n/a	2	3	2.25	n/a	1	n/a
Vascular endothelium	24[b]	0	3	n/a	3	n/a	0	n/a	3	n/a	3	n/a	0	n/a	1	n/a	1.8	n/a	2	n/a
Vascular smooth muscle	5	0	0	n/a	0	n/a	0	n/a	3	n/a	3	n/a	0	n/a	1	n/a	1.4	n/a	1	n/a
Fibrovascular stroma	7	10	2	3	1.86	2.6	1	0	3	3	3	3	0	0	1	2	1.8	1.5	2	1
Bone (osteosarcoma)	0	1	n/a	3	n/a	n/a	n/a	n/a	n/a	3	n/a	n/a	n/a	n/a	n/a	3	n/a	n/a	n/a	n/a
Cardiac muscle	1	0	0	n/a	n/a	n/a	n/a	n/a	3	n/a	n/a	n/a	n/a	n/a	3	n/a	n/a	n/a	n/a	n/a
Skeletal muscle	1	0	0	n/a	n/a	n/a	n/a	n/a	2	n/a	n/a	n/a	n/a	n/a	2	n/a	n/a	n/a	n/a	n/a
Structural smooth muscle	8	0	0	n/a	0.375	n/a	1	n/a	3	n/a	3	n/a	0	n/a	1	n/a	1.47	n/a	1	n/a
Endometrial glands	1	1	3	3	n/a	n/a	n/a	n/a	3	3	n/a	n/a	n/a	n/a	3	3	n/a	n/a	n/a	n/a
Myometrium	1	0	2	n/a	n/a	n/a	n/a	n/a	2	n/a	n/a	n/a	n/a	n/a	2	n/a	n/a	n/a	n/a	n/a
Germ cells	1	1	0	0	n/a	n/a	n/a	n/a	0	3	n/a	n/a	n/a	n/a	2	3	n/a	n/a	n/a	n/a
Interstitial (Leydig) cells	1	0	0	n/a	n/a	n/a	n/a	n/a	3	n/a	n/a	n/a	n/a	n/a	3	n/a	n/a	n/a	n/a	n/a
Sertoli cells	1	0	0	n/a	n/a	n/a	n/a	n/a	3	n/a	n/a	n/a	n/a	n/a	3	n/a	n/a	n/a	n/a	n/a
Endocrine pancreas	1	0	2	n/a	n/a	n/a	n/a	n/a	3	n/a	n/a	n/a	n/a	n/a	3	n/a	n/a	n/a	n/a	n/a
Peripheral ganglia	1	0	3	n/a	n/a	n/a	n/a	n/a	3	n/a	n/a	n/a	n/a	n/a	3	n/a	n/a	n/a	n/a	n/a
Cerebral neurons	1	0	0	n/a	n/a	n/a	n/a	n/a	2	n/a	n/a	n/a	n/a	n/a	2	n/a	n/a	n/a	n/a	n/a
Cerebellar granular layer	1	0	1	n/a	n/a	n/a	n/a	n/a	3	n/a	n/a	n/a	n/a	n/a	3	n/a	n/a	n/a	n/a	n/a
Cerebellar Purkinje cells	1	0	0	n/a	n/a	n/a	n/a	n/a	0	n/a	n/a	n/a	n/a	n/a	2	n/a	n/a	n/a	n/a	n/a

[a]hMito has only one normal squamous epithelium sample

[b]hMito has only 15 endothelium samples; positivity was less than 60% in 9 samples

Abbreviations: TMA, tissue microarray; hHLA-A, human HLA-A; hKu-80, human Ku80; hMito, human mitochondria; IQR, interquartile range
